# An Experimental Study on Drilling Behavior of Silane-Treated Cotton/Bamboo Woven Hybrid Fiber Reinforced Epoxy Polymer Composites

**DOI:** 10.3390/polym15143075

**Published:** 2023-07-18

**Authors:** Karthik Aruchamy, Sathish Kumar Palaniappan, Rajeshkumar Lakshminarasimhan, Bhuvaneshwaran Mylsamy, Satish Kumar Dharmalingam, Nimel Sworna Ross, Sampath Pavayee Subramani

**Affiliations:** 1 Department of Mechatronics Engineering, Akshaya College of Engineering and Technology, Coimbatore 642109, Tamil Nadu, India; 2 Department of Mining Engineering, Indian Institute of Technology, Kharagpur 721302, West Bengal, India; 3 Department of Mechanical Engineering, KPR Institute of Engineering and Technology, Coimbatore 641407, Tamil Nadu, India; 4 Department of Mechanical Engineering, K. S. R. College of Engineering, Tiruchengode 637215, Tamil Nadu, India; 5 Department of Mechanical Engineering, Saveetha School of Engineering, SIMATS University, Chennai 602105, Tamil Nadu, India; 6 Department of Mechanical Engineering, K. S. Rangasamy College of Technology, Tiruchengode 637215, Tamil Nadu, India

**Keywords:** cotton/bamboo woven fabric, silane treatment, thrust force, SEM analysis, RSM, machining operation

## Abstract

Machining is considered to be an important post-manufacturing process. Evaluation of machinability of natural-fiber-reinforced composites is important owing to its wide application spectrum. Current experiments focus on the drilling parameters of cotton/bamboo woven fabric reinforced epoxy composites laminates using a solid twist drill. Composites were manufactured with 45 wt.% cotton/bamboo woven fabric in epoxy resin using a compression molding method. Drilling experiments were carried out in pillar-type drilling machine and the drilling characteristics, such as thrust force, were analyzed using four process parameters like spindle speed, feed rate, drill diameter, and silane-treated fabric. Drilling experiments were carried out using the Box–Behnken Experimental Design, and the recommended drilling characteristics were analyzed using quadratic models based on response surface methodology. It was observed from the results that the thrust force is low with small drill-bit diameter, higher cutting speed, and lower feed rate, according to the response surface analysis. Surface morphology of the drilled hole suggested that a better quality of hole can be obtained at lower feed rates.

## 1. Introduction

The study of composite material from the last two decades that focuses on investigating natural-fiber-reinforced composite is an important branch of materials science [1,2,3,4,5]. Cotton, bamboo, bark, wood, pulp, cereal straw, bark, bagasse, corncobs, nut shells, and vegetable (e.g., coir, ramie, sisal, jute, flax, sun hemp, banana, and pineapple) are the examples of plant-based natural fibers [6,7,8]. The major microconstituents of these fibers are hemicelluloses, cellulose, wax, and lignin, along with a minimum percentage of extractives. The composition of fiber varies depending on their origin [9,10,11]. The fibers used in engineering applications mainly include synthetic fibers and natural fibers. Synthetic fibers such as carbon fiber and glass fiber have been widely used in different fields because of their lightweight and high strength, excellent mechanical properties, corrosion resistance, fatigue resistance, and so on. The above advantages can make synthetic fiber composites the main load-bearing components in engineering applications [12,13,14]. Also, natural fibers show several benefits compared to standard glass and carbon fibers. Natural fibers are of low cost, abundantly available, environment-friendly, easy to process, have low density, and possess reasonably good flexural and tensile modulus. By considering some key problems in synthetic fibers such as high cost, environmental problems during the preparation, and nonrenewable material, natural fibers overcome the above disadvantages and can be mixed with polymer matrix to create composites. Even if the strength of the fiber-reinforced composites is sometimes lower than the individual fiber itself, either hybridization or suitable treatments may eradicate this. However, the strength of composites, at most times, would be less than the individual fiber itself. Natural fibers with biodegradable and renewable properties make composting and incineration easier than all other synthetic and industrial fibers. In addition, the fibers contain stored atmospheric carbon dioxide and have a minimum embodied energy than industrially produced glass fibers [15,16,17].

Even though natural fibers have the possibility to complement synthetic fibers in polymer composite materials [15,16], natural fibers have limitations in terms of mechanical performance and absorption of humidity [17,18]. Regardless of whether thermoplastic or thermoset polymers are utilized as the matrix material, the same limitations exist. According to previous studies [4,19], the chemical incompatibility between the hydrophilic lignocellulosic molecules of the natural fiber and the hydrophobic thermoplastic molecules is the key factor in limiting the mechanical characteristics of natural-fiber-reinforced thermoplastic composites. Because of this incompatibility, achieving good fiber matrix interface bonding is challenging, resulting in inadequate load shift between the reinforced fibers and matrix. In order to obtain the requisite compatibility between the fiber and matrix, various solutions have been suggested, including chemical treatments of lignocelluloses fibers and the use of compatibilizers. Various authors stated that the different chemical processes can be used to enhance the lignocellulosic fiber compatibility with polymer matrices. The use of alkali treatment, isocyanate treatment, acetylation, permanganate treatment, peroxide treatment, benzoylation, and silane treatment were all studied. Treatments such as alkaline and silane were frequently reported among the various approaches [18,19].

The fibers were soaked in alkaline solution, most often NaOH, for a specified period of time during the alkaline treatment. It was expected to improve the mechanical bonding by enhancing the roughness of fiber surface. It increases the crystallinity of the fiber which enhances the possibility of chemical interaction with the matrix [20,21,22]. It also exposes more reactive functional groups of cellulose to be bonded with a polymer matrix. Alkaline treatment effects were studied by some authors on kenaf fiber [20]. Cleaning the fiber surface with an alkaline treatment using a 6% NaOH solution was found to be effective. On the other hand, a 9% NaOH solution was found to degrade the fiber surface and decreased its strength. The fibers are normally soaked in silane solution diluted in water/alcohol. As a result, silane hydrolyses itself into silanol from alcohol that contains water. The silanol combines with the OH groups of cellulose in natural fibers to generate stability in covalent linkages with cell walls that assimilate onto the fiber surface [21]. Silane treatment increases the cross-linking in the connected regions between fiber and the matrix, allowing for better interaction between the fiber and matrix [22]. Mechanical properties such as tensile strength and modulus of flax fiber reinforced with epoxy composite were found to increase, as stated by Van de Weyenburg et al. [23]. Results showed that improvement in the tensile strength and modulus was roughly 30% and 45%, respectively, when 40 vol.% of treated flax fibers were utilized as a reinforcement in epoxy matrix. This improvement was due to the combined chemical treatment of flax fibers, i.e., by using a combination of sodium hydroxide and silane coupling agent chemicals.

Natural-fiber-reinforced polymer composites are more difficult to machine because of lack of machining records and a higher level of parameter complexity [24]. Occurrence of voids, delamination, matrix, and fiber debonding are some of the examples for manufacturing and machining faults. Despite the fact that the majority of fiber-based hybrid composites are made to actual shape by using the machining techniques such as drilling, grinding, and milling, which are completely unavoidable at the time of assembly process, drilling is considered the most important machining techniques in the overall process. When compared to drilling of conventional metals and alloys, the drilling procedure for fiber-reinforced composite samples is more complex because of the anisotropic nature of samples. Due to the presence of insoluble constituents in fiber-reinforced composites, the isotropy cannot be defined as in conventional metals and alloys, which makes them more anisotropic materials. This complexity is considered to be a significant one in the drilling of fiber-reinforced composites. It finds its application in structural automobile components, kitchen cabinets, doors, furniture, electronic components, and so on. The end product is assembled with sliced composite panels to form the desired shape and dimensions, and attaching them with bolts and screws in all of the above applications. As a result, before placing it into use, drilling is performed to complete the assembly process. Hence, drilling is considered to be extremely important, because a fault in the structural integrity of the drilling process influences the quality of the final product [25].

Drilling experiments in fiber-based polymer composites have sufficiently provided an indication of the importance and research direction. On a coir–polyester composite, the influence of drill diameter, speed, and feed on torque, thrust force, and tool wear was investigated. Drilling studies on roselle–sisal hybrid composites were performed and optimized using an artificial neural network model [26,27]. Few experimenters investigated initially with peel-up delamination and later with push-down delamination for hemp/flax-fiber-based composites and also discovered that the most important elements to consider throughout the drilling process are feed rate and cutting speed [28]. In the drilling of sisal polypropylene composites, drill geometry was determined to be the most influential element [29,30,31]. Drilling was performed on a cotton/bamboo (CB) woven fabric reinforced composite, and thrust force was calculated for various combinations of cutting speed, feed rate, and drill shape. The cutting parameters, as well as the input parameters, were optimized using a Box–Behnken Experimental Design (BBED). When the drill diameter was increased, the results revealed that the feed rate increases with the decrease in cutting speed. Drill diameter and rate of feed influence the increment of thrust force, according to Srinivasan et al. [32]. Using BBED and analysis of variance (ANOVA), the effect of thrust force was investigated, and they observed that low feed rate, high spindle speed, and smaller drill diameter resulted in the lowest thrust force.

In spite of various studies on drilling behavior of natural fiber composites, only few studies discuss the machining behavior of treated woven fiber reinforced composites. In the current study, silane-treated CB reinforced hybrid composites were analyzed for their machinability behavior through drilling process. Cutting thrust force was evaluated with various feed rates, cutting speeds, and drill geometries. The optimum parameter was determined in order to achieve the lowest cutting thrust force by using the response surface methodology (RSM) technique. The research experiments were controlled by BBED. The data were analyzed using 3D plots and influence graphs. The drilled surface morphology was examined using a scanning electron microscopy (SEM) and the optimal parameter settings for obtaining a good quality hole and better machinability were recommended.

## 2. Materials and Methodology

### 2.1. Fabrication of CB Woven Fabric

In this paper, the CB woven fabric composites were manufactured with cotton yarn and bamboo yarn packed with corresponding warp and weft directions. In this study, cotton yarn, bamboo yarn, and CB woven fabric were collected from Pallava Spinning Mills (Pvt.) Limited, Erode District, Tamil Nadu, India, and Ganapathi Chettiar Tex, Tirupur District, Tamil Nadu, India, with good-quality yarn that has bamboo fiber length of 36 mm, fiber fineness of 1.52 dtex, linear density of 0.155 tex, moisture regain value of 11.42%, tenacity of 22.84 g/tex, and elongation of 21.2% [33]. The yarn and fabric particulars are given in Table 1 and Table 2, respectively.

### 2.2. Silane Treatment of CB Fabrics

CB fabrics were immersed in the 1.5% concentrations of 3-aminopropyl trimethoxy silanes solution (procured from M/s. Covai seenu, Coimbatore, Tamil Nadu, India) with water and acetone (50/50 volume) for 2 h. From various screening experiments and based on the literature, the concentration of silane was taken as 1.5% [23]. The fibers were then soaked in 1.5% silane mixed with 2% acetic acid in the form of acetic anhydride for pH adjustment between 4.5 and 5, and sundried for 24 h. Then, the fibers were oven dried at 80 °C for 4 h to maintain their stiffness. Silane coupling agents were expected to effectively modify the natural fiber–polymer matrix interface and increase their interfacial strength.

### 2.3. Fabrication of Composite Laminates

Composite laminates were manufactured using a compression molding technique. Initially, wax was applied to the surface of the mold for easy removal, and the size of the mold measured 270 mm × 270 mm × 3 mm. The CB woven fabrics (2 layers each) were laid and arranged, layer after layer, with epoxy adhesive to keep woven fabrics composed. For this experiment, Araldite LY 556 epoxy resin and HY951 hardener (procured from M/s. Covai seenu, Coimbatore, Tamil Nadu, India) were used. To assist gradual and homogeneous curing of the resin, a 10:1 mix of resin and hardener was used. A composite plate was created by stacking alternate layers of resin and fabric. It was then compressed at 30 MPa for 1 h at 160 °C. Although a greater temperature will speed up the process of curing, the natural fiber’s existence limited high-temperature processing. A consistent weight fraction of 45% was used to make the composite laminates. The composite included isotropic character, which is the most important requirement in various commercial applications and is the only way to ensure uniform load-carrying capability of the material.

### 2.4. Drilling Process Parameters and Optimization

**Figure 1:**
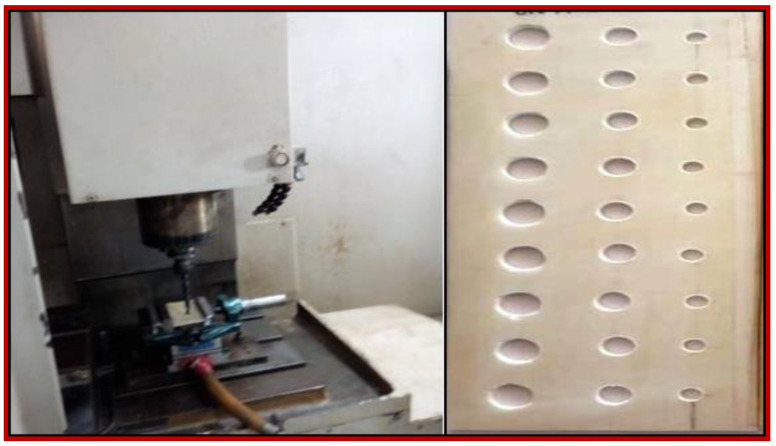
Drilling of CB composite laminate.

Drilling operations were performed in a pillar-type drilling machine (*Make: Kirloskar, Pune, India*) using HSS drill bits with pointed ends. All the operations were carried out at ambient atmospheric conditions. In this experimental work, data acquisition systems were used along with a drill dynamometer to obtain thrust force signals. Forces developed on the drill bit axis or spindle axis during the machining process are termed as thrust force, which induces damage during the drilling of silane-treated woven CB composite laminates, as shown in Figure 1.

The hole-making process in composite materials is influenced by a number of variables such as spindle speed, drill diameter, drill type feed rate, drill point angle drill material, type of matrix, type of fiber reinforcement, and fiber volume fraction. These drilling parameters define and influence the thrust force, surface quality of the drilled hole, delamination near the drilled hole, and other geometrical factors such as roundness and eccentricity [34]. Many investigations have discovered that the major influencing parameters in the drilling of fiber-reinforced composite panels include feed rate [35], drill diameter [36,37], and cutting speed [31]. Drilling operations were carried out by changing the drill bit rotation speed (rpm), drill diameter (mm), and rate of feed (mm/min). Table 3 shows the various process parameters used for the drilling of treated CB fabric woven hybrid composites.

A scanning electron microscope (model: VEGA 3 TE SCAN) was used to study the surface topography of the drilled holes in silane-treated CB composite laminates. A thin layer of sputtered gold was coated on the surface of all the tested composite specimens to improve electrical conductivity and to obtain better images.

RSM is the collection of mathematical and statistical data to develop modeling and analyses of engineering problems [38]. Nowadays, RSM is used in the design of experiments (DOE). Also, RSM helps in identifying the correlation between input parameters to obtain the response surface. Box and Draper [39] developed a model to suit physical experiments and later for machining problems. Further, RSM also has its objective to later mine the optimization of process parameters.

Douglas C. Montgomery [40] also developed a model relating the process variables with output response, which was utilized for process prediction and control. The experiment domain’s boundary must be investigated in RSM. The process parameters for this analysis were feed rate, cutting speed, and diameter of the drill, and BBED was utilized to formulate the design of experiments. BBED, an alternate way to central composite design (CCD), is the common statistical tool employed to frame the number of experiments. This approach was used to analyze experiments comprising three levels of design. The BBED can be rotated, and it is said to have no fractional factorial design, which makes it easy to understand the results [41]. In RSM, the general correlation of the control parameters and their responses is illustrated in Equation (1):

(1) $$Y=\beta_{0}+\beta_{1}x_{1}+\beta_{2}x_{2}+\beta_{i}x_{i}+\ldots \ldots +\varepsilon$$

where Y is the response (thrust force), β_0_, β_1_, β_2_,…β_i_ are the regression coefficients, x_1_, x_2_,…x_i_ denote the predictor variable, namely, feed rate, diameter, and cutting speed, and ε shows the error occurring in the thin model. Generally, in RSM, quadratic response functions (Equation (2)) are used and are represented as follows:

(2) $$Y=\beta_{0}+\sum_{i=1}^{k}\beta_{1}x_{1}+\sum_{i=1}^{k}\beta_{ii}x_{i}^{2}+\sum_{i<j}^{k}\beta_{ij}x_{i}x_{j}+\varepsilon$$

## 3. Results and Discussion

### 3.1. Thrust Force Analysis

**Figure 2:**
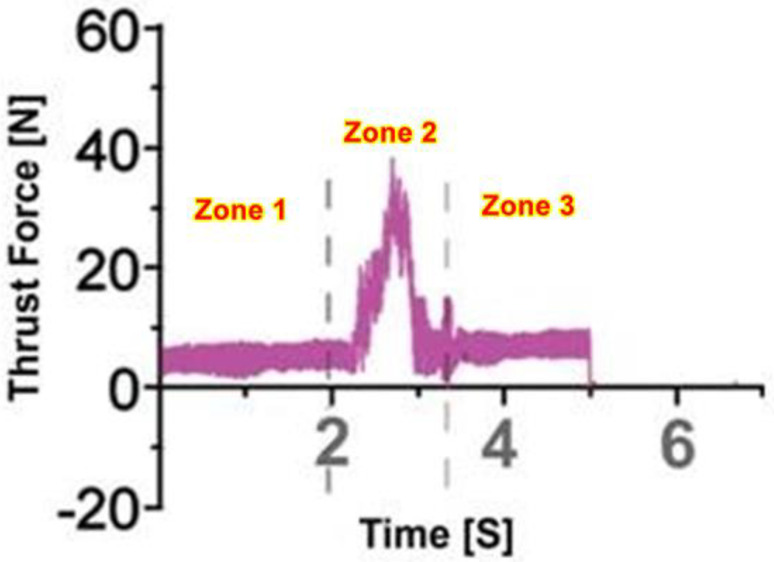
Average cutting forces signal that used a twist drill at 1800 rpm and 60 mm/min.

Silane-treated CB woven fabric composites were drilled using twisted drill bits, and the thrust force signals were obtained, as shown in Figure 2. The plot was obtained during drilling at a speed of 1800 rpm and at a feed of 60 mm/min. This plot depicts different phases, as shown in the figure: initially, the thrust force was found to be low before the entry of the drill bit and during the progress of the drill bit. Next, the thrust force increased to the maximum level during the process of making the hole, i.e., full contact of the drill bit and the specimen. Then, as the drill bit reached the bottom of the specimen, it gradually reduced the thrust force and marked the exit of the drill bit where the thrust force finally attained a zero value [37].

### 3.2. Response Surface Methodology (RSM)

By considering input variables, a quadratic equation was newly developed for determining thrust force in order to attain exact process parameters. The final model for thrust force is

(3) $$\begin{array}{ll} Y(N)=19.9525 & +\;(4.072667\times d)-(0.30465\times f)+(0.004042\times n)-(0.03583\times d\times f)\\ & -\;(0.00049\times d\times n)+(0.000183\times f\times n)-(0.06561\times d^{2})+(0.007761\times f^{2})-(3.5Ε\\ & -\;6\times n^{2}) \end{array}$$

Table 4 shows the experimental results for the drilling of CB woven fiber reinforced hybrid epoxy composites using BBED.

Table 5 shows the ANOVA table for thrust force. Based on F and *p*-value, the RSM model can be simultaneously analyzed, considering all the design factors and the value of F, which predicts the quality of the entire design model. The very low probability factors identified by *p*-value have an insignificant effect on the response [42]. The higher F value and lower *p*-value (*p*-value < 0.05) significance enhanced the fit for the predicted designed model with the experimental model. The experimental result was calculated with a confidence level of 95%. Any value of *p* less than 0.05 indicates that the developed thrust model will be significant. However, the F value of the lack of a fitted model is 0.372, which indicates that the model is insignificant. It proves that the planned model is very suitable for the prediction of thrust force during drilling of developed hybrid composites [43]. In Table 5, it is shown that the feed rate (f) and spindle speed (N) are significant. The coefficient correlation (R-Seq) and adjusted coefficient correlation (0.98438 and 0.9643) indicate that the model is sufficient, and so the procedures were successfully utilized to find the cutting thrust force during the drilling process in composite laminates.

**Figure 3:**
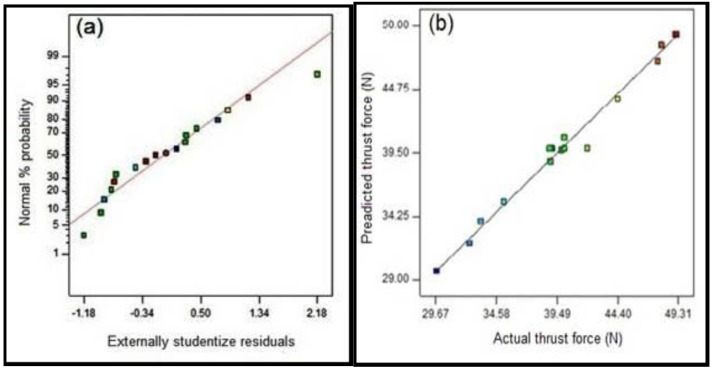
(**a**) Normal probability of residuals. (**b**) Predicated thrust force in the drilling of the composites.

Figure 3a depicts the normal percentage of probability plot with respect to internally studentized residuals and it is observed from the graph that the result is grouped to form a straight line; hence, it confirms the efficiency of the developed model. Similar results were obtained by some of the researchers previously [44]. The association between the actual and predicted thrust is depicted Figure 3b. This result shows that the predicted values are in close agreement with the experimental values [35].

**Figure 4:**
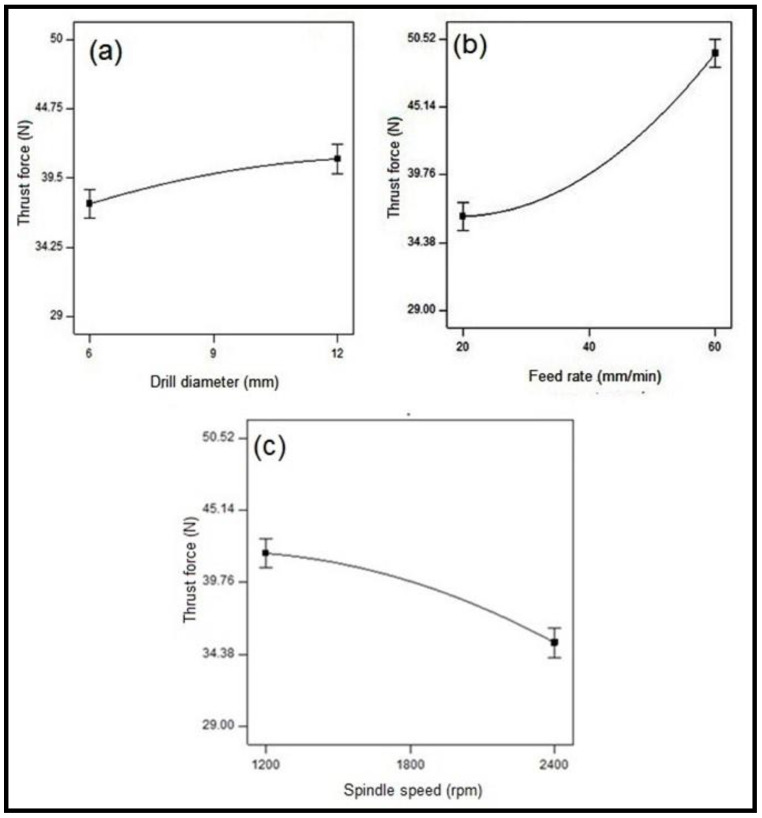
Influence of machining characterization on thrust force in CB fabric/epoxy composites.

Figure 4 shows the interrelationship between the considered process parameters and the cutting thrust force of CB-reinforced epoxy hybrid composite laminates. Figure 4a explains the influence of the drill diameter on the thrust force. It turns out that as the diameter of the drill bit increases, the thrust force also increases [42].

As the diameter of the drill bit increases, the contact area between the drill bit and the laminate becomes larger; hence, due to higher heat generated, the thrust force increases. The influence of feed speed on thrust is depicted in Figure 4b. The results show that whenever the rate of feed increases, the thrust force also increases. As the diameter of the drill (d) and the feed (f) increases, the cross-sectional area (A) increases because of the formed chip area, A = df/4, and the thrust force also increases, so the axial thrust and the resistance to the developed chip increase [45]. Figure 4c shows that the increase in spindle speed decreases the thrust force, and due to higher spindle speed, the chip formation softens, thereby minimizing the heat of the material and increasing the thrust force. It could be stated from the study that the change in cutting speed and feed rate influenced the thrust force significantly, irrespective of the working environment [46].

**Figure 5:**
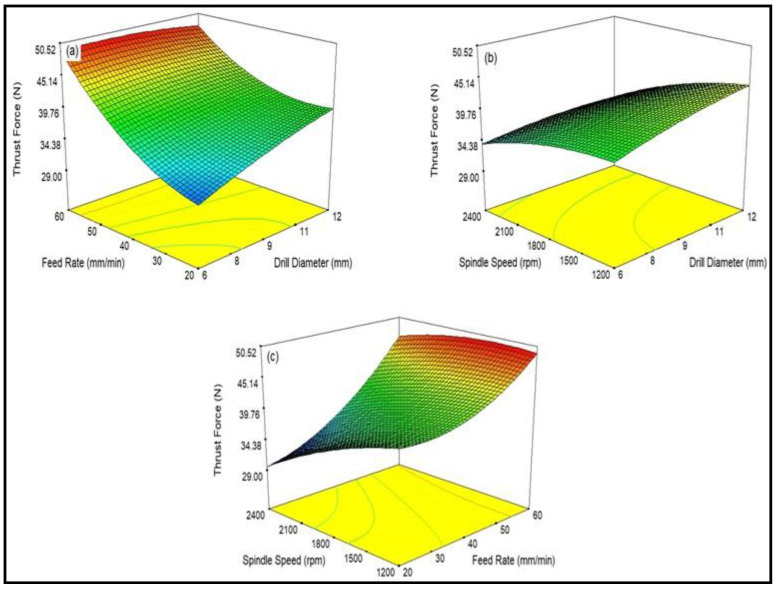
A 3D response plot for thrust force during drilling of silane-treated CB fabric/epoxy composites; (**a**) influence of drill diameter and feed rate on thrust force; (**b**) influence of drill diameter and spindle speed on thrust force; and (**c**) influence of feed rate and spindle speed on thrust force.

Figure 5 shows the 3D model of the thrust force during the drilling of silane-treated CB composite laminate. The effect of the reaction among processing variables is discussed through 3D response plots. The 3D model has a graph that shows the influence of two changing variables while maintaining the constant third parameter throughout the experiments. Figure 5a indicates the variation of thrust force with the diameter of the drill and the rate of feed, keeping the cutting speed constant. The results show that the thrust force increases as the diameter of the drill bit and feed rate increase. Figure 5b shows the impact of drill diameter and cutting speed on thrust force. As a result, it was found that as the spindle speed of the CB composite laminate increased, the thrust force decreased. Figure 5c explains the plot of cutting speed and feed rate on the thrust force of the CB composite laminate. The findings show that the enhancement in feed and speed increased the thrust force of the CB composite laminate. It can be seen from the experiment that the drill diameter, feed rate, and cutting speed affected the machining operation irrespective of the working environment.

### 3.3. Comparison of Experimental and Thrust Force Values

**Figure 6:**
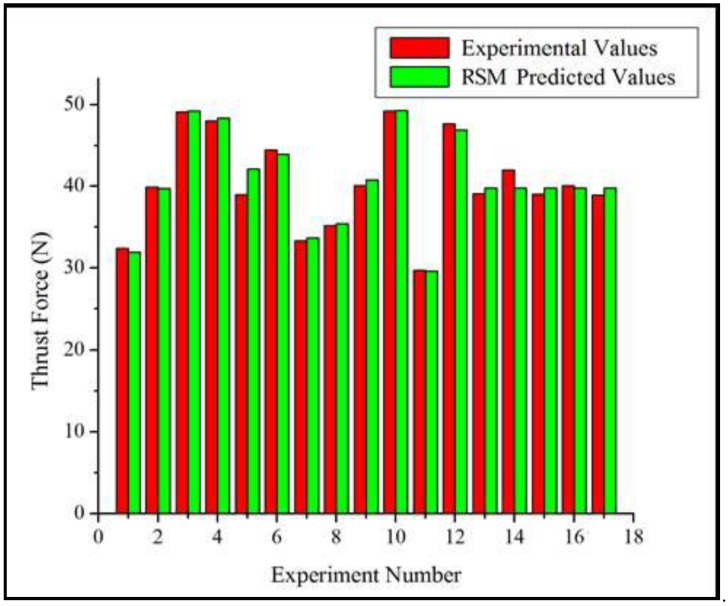
Comparison of experimental and RMS thrust force values.

In this experiment, the R-squared value of the model is greater than 90%, and so the modeling system can be effectively used to predict the different properties of composite drilling. The comparison between the RSM value and the experimental value is shown in Figure 6. It is proven that the relevant statistics between the experimental model result and the RSM predicted result can be obtained through the RSM technique.

### 3.4. SEM Observation

**Figure 7:**
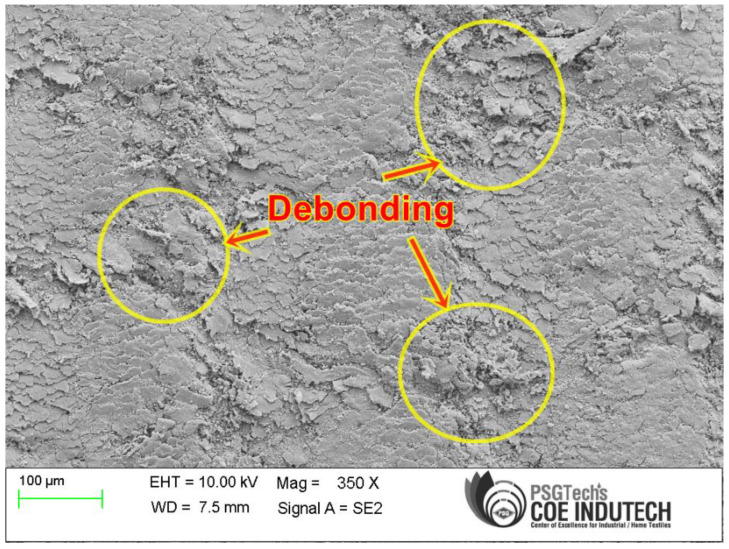
SEM image of drilled hole drill (1800 rpm and 60 mm/min).

**Figure 8:**
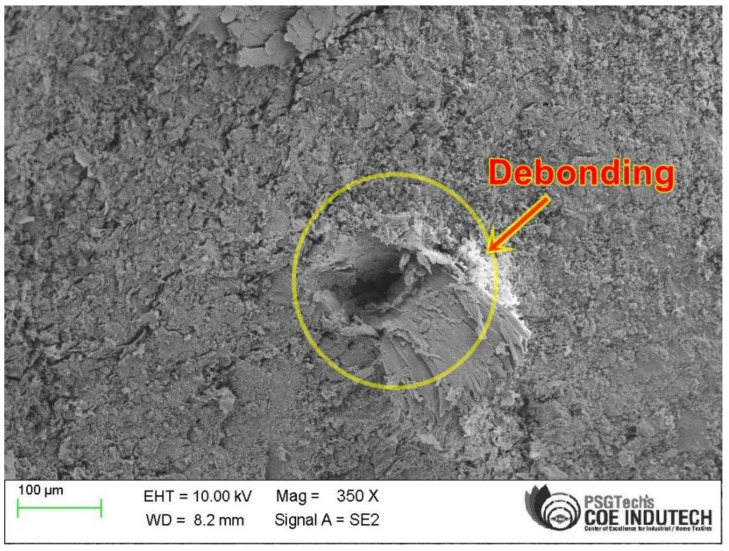
SEM image of drilled hole drill (2400 rpm and 20 mm/min).

The morphology of the drilled holes in silane-treated CB composite laminates was examined using a scanning electron microscope at various locations. The SEM micrograph shows the microstructure of the drilled surface. It could be observed that debonding between fiber and matrix, surface roughness, and fiber pull-out exist in the drilled silane-treated CB fabric epoxy composite laminate using a solid twist drill. Figure 7 shows that minimum cutting speed and maximum feed rate damage the drill walls and cause large fiber pull-out and surface roughness. This is due to the effect of high thrust force applied on the drill zone of the composite laminate [47]. Figure 8 shows the morphology of the composite specimen under better cutting behavior with less drill damage during drilling when compared to high-speed cutting and minimum feed rate conditions. This is due to less contact of the drill bit with the CB composite laminates. The result indicates that the thrust force is low and has a better quality of hole at lower feed rates [33,48].

## 4. Conclusions

Cotton/bamboo woven fabric reinforced epoxy composite laminates were manufactured using a compression molding technique and were subjected to machinability analysis. The following conclusions were obtained from the experimental study.

- From the thrust force analysis, it could be observed that during drilling, three significant phases of thrust force could be obtained. The maximum value of thrust force was during the complete contact of drill bit with the composites.
- The influence of various process parameters on the thrust force on fabric/epoxy composite can be examined by RSM. The optimum cutting parameters are identified at a minimum rate of feed (20 mm/min), maximum spindle speed (2400 rpm), and drill diameter (6 mm). The adequacy of this model is proven to be fitted for finding thrust force on the composite material.
- The size of the hole is one of the main factors and it turns out to be the reason for the increase in thrust force. When the thrust force increases, the size of the hole also increases considerably and the composite laminate thickness plays a vital role in thrust force development.
- From the response plots and 3D surface plots, it can be concluded that the thrust force increases with drill diameter and feed rate and decreases with spindle speed. Hence, lower feed rate and small drill diameter at higher spindle speed produce good-quality holes.
- From the SEM micrograph, the minimum thrust forces developed at a minimum feed rate and high cutting speed along with minimum diameter of the drill is proven. Hence, it can be concluded from the study that the woven cotton/bamboo fabrics can be machined with lower feed rates and drill diameter but at higher speeds. Such composites can be applied in low and medium load-carrying structural applications in automobile, aerospace, and marine industries, where machining of the composites is an integral part of manufacturing.

## Figures and Tables

**Table 1:** Particulars of yarns.

S. No.	Properties	Cotton Yarn	Bamboo Yarn
1	Fiber length (mm)	40	36
2	Fiber fineness (dtex)	1.35	1.52
3	Linear density (tex)	0.115	0.155
4	Moisture regain value (%)	7.85	11.42
5	Tenacity (g/tex)	32.22	22.84
6	Elongation (%)	5.8	21.2

**Table 2:** Particulars of CB woven fabric [30,31].

S. No.	Fabric Particulars	Values
1	Fabric GSM (g)	164
2	End per inch (EPI)	69
3	Picks per inch (PPI)	54
4	Thickness (mm)	0.38

**Table 3:** Drilling process parameters [30,31].

S. No.	Process Parameter	Unit	Upper Limit	Lower Limit
1	Cutting speed (n)	rpm	3000	1000
2	Drill diameter (d)	mm	12	6
3	Feed rate (f)	mm/min	60	20

**Table 4:** The experimental results for the BBED.

S. No.	Run	Diameter of the Drill (mm)	Feed Rate (mm/min)	Speed of the Spindle (rpm)	Thrust Force of Cutting (N)
1	2	6	20	1800	32.43
2	16	12	20	1800	39.89
3	11	6	60	1800	49.11
4	12	12	60	1800	47.97
5	14	6	40	1200	38.99
6	10	12	40	1200	44.43
7	7	6	40	2400	33.34
8	4	12	40	2400	35.22
9	15	9	20	1200	40.11
10	1	9	60	1200	49.23
11	8	9	20	2400	29.75
12	17	9	60	2400	47.67
13	3	9	40	1800	39.12
14	13	9	40	1800	41.98
15	9	9	40	1800	39.06
16	5	9	40	1800	40.11
17	6	9	40	1800	38.91

**Table 5:** Analysis of variance (ANOVA) table for thrust force.

Source	d_f_	Adj SS	Adj MS	F Value	*p*-Value Prob > F
Regression model	9	535.903	59.545	49.041	<0.0001
d	1	23.256	23.256	19.154	0.0032
f	1	335.405	335.405	276.236	<0.0001
n	1	89.646	89.646	73.832	<0.0001
d_f_	1	18.490	18.490	15.228	0.0059
d_n_	1	3.168	3.168	2.609	0.1503
n_f_	1	19.360	19.360	15.945	0.0052
d^2^	1	1.468	1.468	1.209	0.3079
f^2^	1	40.581	40.581	33.422	0.0007
n^2^	1	6.584	6.584	5.423	0.0527
Residual	7	8.499	1.214		
Lack of fit	3	1.855	0.618	0.372	0.7784
Pure error	4	6.644	1.661		
Cor. total	16	544.402			
Std. Dev.		1.102	R-Squared	0.984	
Mean		40.431	Adj R-Squared	0.964	
C.V.%		2.725	Pred R-Squared	0.926	
Press		40.065	Adeq Precision	23.245	

## Data Availability

The data presented in this study are available on request from the corresponding author. The data are not publicly available due to privacy.
